# Supplementary material for: ER – lysosome contacts at a pre-axonal region regulate axonal lysosome availability
Source: Nat Commun. 2021 Jul 23;12:4493. doi: 10.1038/s41467-021-24713-5 (PMC8302662; doi:10.1038/s41467-021-24713-5)
Supplement: Supplementary file 1 — Supplementary information [file 41467_2021_24713_MOESM1_ESM.pdf]

ER – lysosome contacts at a pre-axonal region regulate axonal lysosome availability

Nazmiye Özkan[1], Max Koppers[1], Inge van Soest[1], Alexandra van Harten[1], Daphne Jurriens[1], Nalan Liv[2], Judith Klumperman[2], Lukas C. Kapitein[1], Casper C. Hoogenraad[1], Ginny G. Farías[1]

Supplementary information includes:

Supplementary Figs. 1-6
Supplementary Table

Supplementary Fig. 1

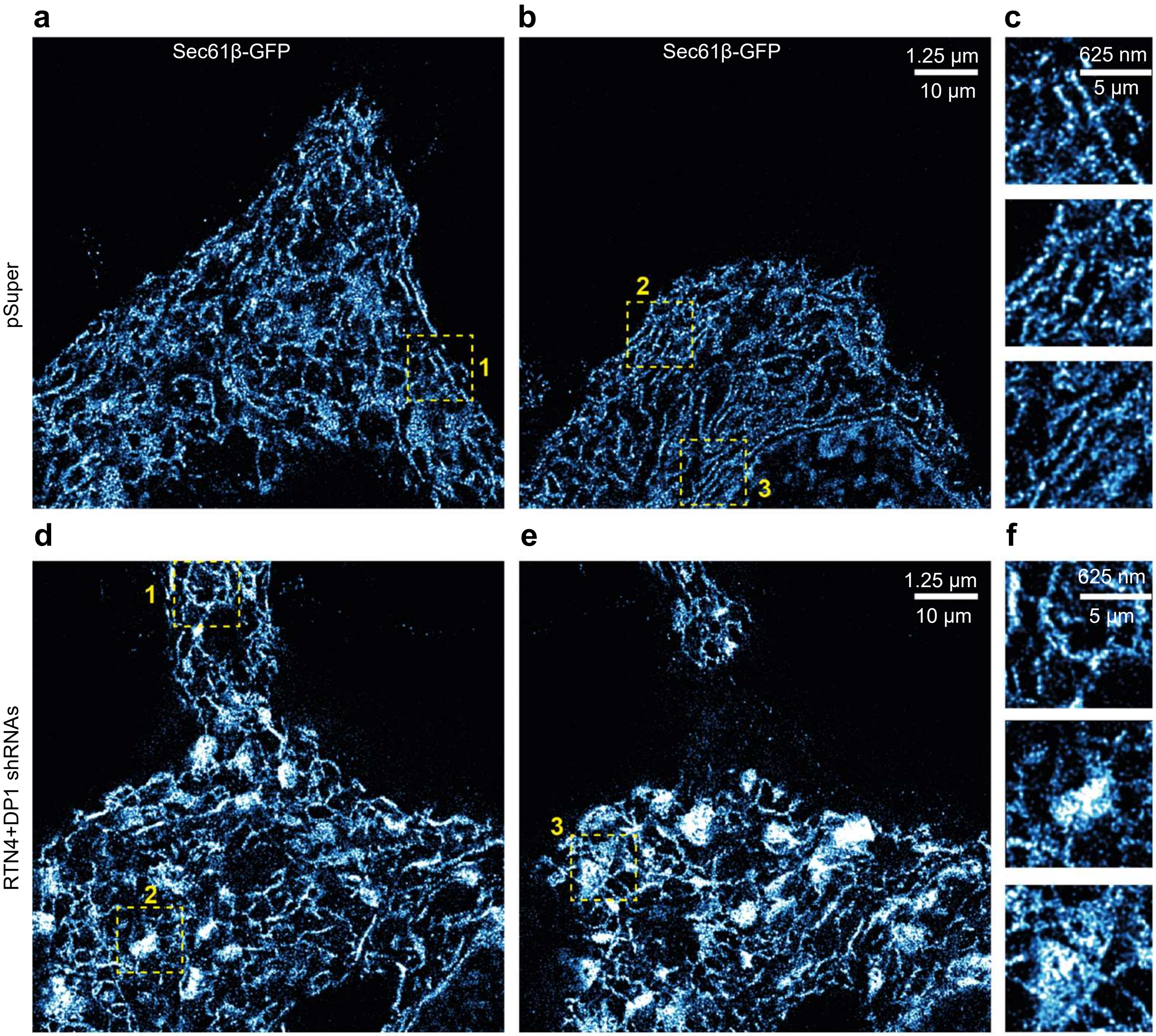

Supplementary Fig. 1 (related to Fig. 1): ER nanoscale structure is altered by knockdown of RTN4 and DP1.

Representative images of nanoscale ER structure from expanded neurons transfected with Sec61β-GFP and a control pSuper plasmid **(a-c)** or pSuper plasmids containing shRNAs against RTN4 plus DP1 **(d-f)** are shown with a full view of a higher plane (**a** and **d**) and a lower plane closer to the coverslip (**b** and **e**). Lower plane contains the start of a dendrite. Zooms from both (**a**, **d**) and (**b**, **e**) (depicted in yellow dotted boxes) illustrating high resolution ER structures are shown in (**c** and **f**). Notice the reduced formation of ER tubules and change of ER shape after knockdown of RTN4 plus DP1 compared to control. Scale bar is 10 μm (**a**), (**b**), (**d**) and (**e**) in the expanded sample. Using the expansion factor this is corrected to estimate the size of structures pre-expansion, being approximately 1.25 μm. Scale bars in (**c**) and (**f**) are 5 μm in the expanded sample and 625 nm after correction for expansion factor.

Supplementary Fig. 2

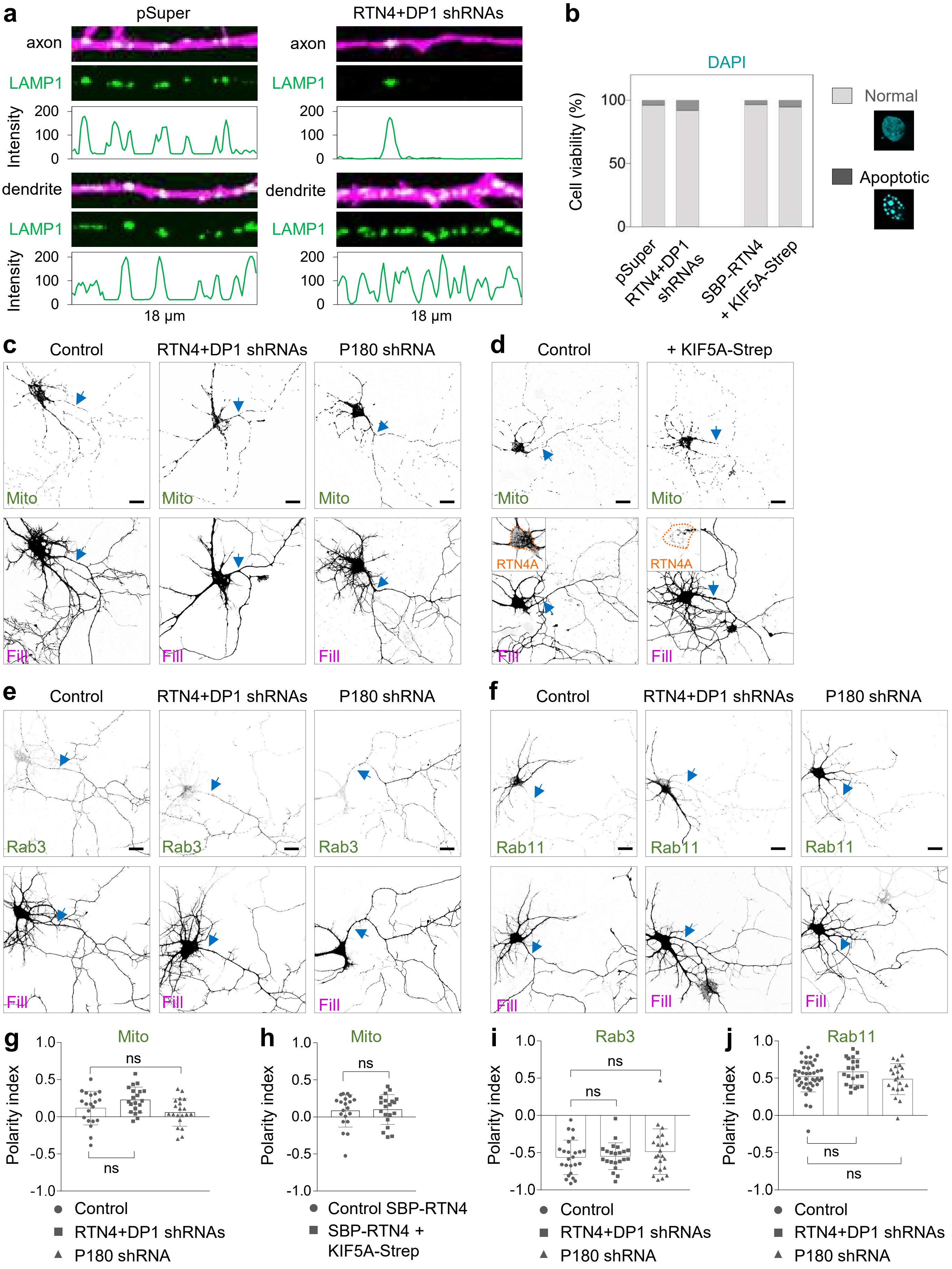

Supplementary Fig. 2 (related to Figs. 1, 2 and 6): ER tubule disruption or somatic ER tubule redistribution into the axon do not alter neuronal viability or distribution of other organelles.

a Representative images of segments of axon and dendrite of DIV7 neurons transfected at DIV3 with a control pSuper plasmid (left panels) or pSuper plasmids containing shRNAs targeting RTN4 plus DP1 (right panels) together with LAMP1-GFP (green) and a fill (magenta). Intensity profile line (bottom).

b Quantification of cell viability of DIV8 neurons transfected at DIV4 with a control pSuper plasmid (n=74) or pSuper plasmids containing shRNAs targeting RTN4 plus DP1 (n=61) or transfected with SBP-RTN4 alone (n=112) or with KIF5A-Strep (n= 145) and labelled with DAPI (cyan) prior to mounting.

c, d Representative images of DIV7 neurons co-transfected with Mito-DsRed and a fill together with a control pSuper plasmid or pSuper plasmids containing shRNAs targeting RTN4 plus DP1 or pSuper plasmids containing shRNAs targeting P180 (**c**), or together with SBP-RTN4 in absence or presence of KIF5A-Strep (**d**).

e, f Representative images of DIV7-8 neurons co-transfected with Rab3-GFP (**e**) or Rab11-GFP (**f**) together with a control pSuper plasmid or pSuper plasmids containing shRNAs targeting RTN4 plus DP1, or pSuper plasmids containing shRNAs targeting P180.

g-j Quantification of mitochondria polarity indices in (**g** and **h**; for conditions shown in **c** and **d**; n=20 per conditions) and quantification of Rab3 and Rab11 polarity indices in (**i** and **j**; for conditions shown in **e,** n=24, n=22 and n=22; respectively and **f**; n=42, n=21 and n=21; respectively). Data are presented as mean values +/- SD and individual datapoints each represent a neuron, in (**g**), (**h**), (**i**) and (**j**); ns-not significant comparing conditions to control (one-way ANOVA test followed by a Dunnet's multiple comparison test) in (**g**) (two-sided Mann-Whitney test) in (**h**), and Kruskal-Wallis test followed by a Dunn's multiple comparison test) in (**i** and **j**). Blue arrows point to the proximal axon and scale bars represent 20 μm in (**c**), (**d**), (**e**) and (**f**). Source data and exact p values are provided as a Source Data file.

Supplementary Fig. 3

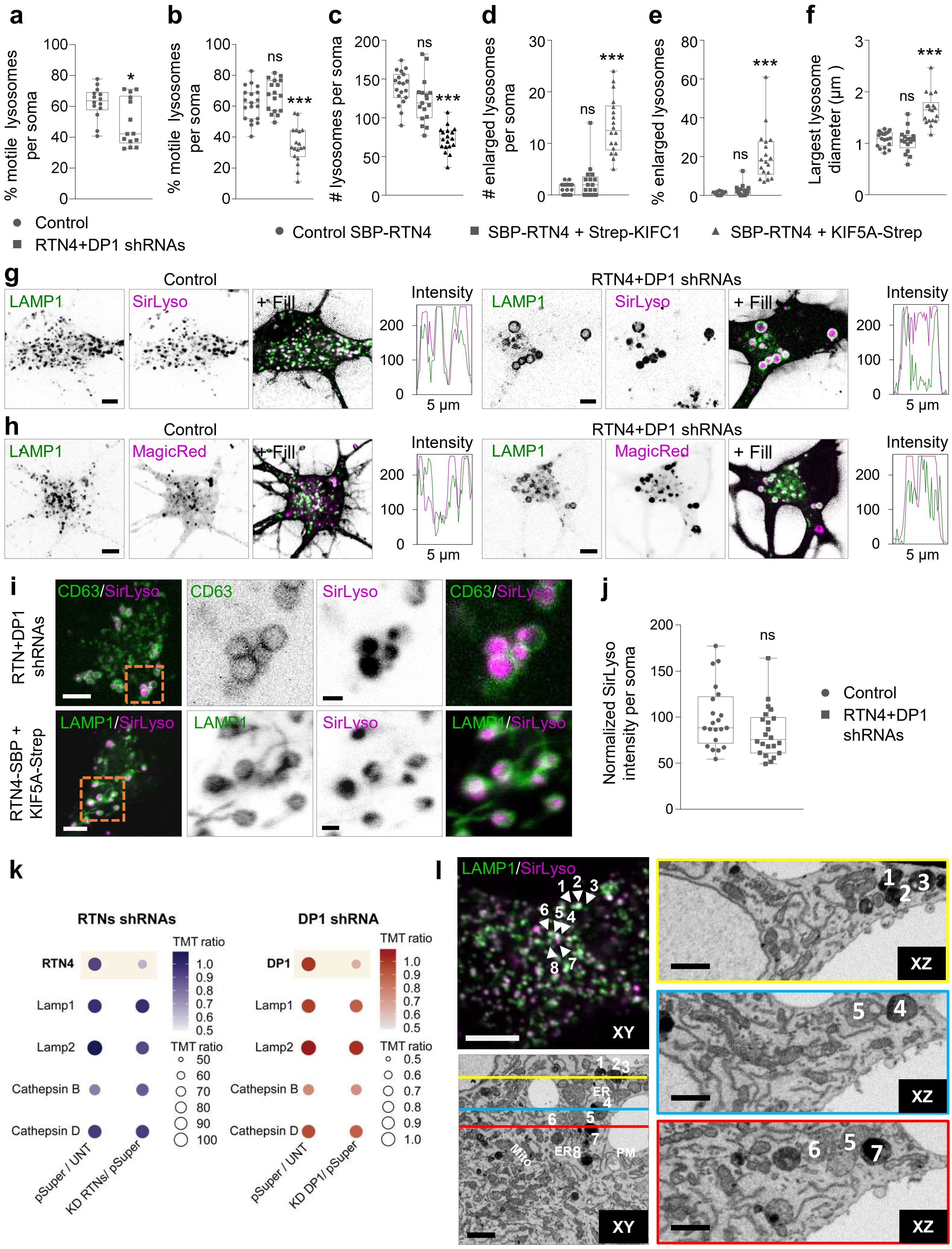

Supplementary Fig. 3 (related to Fig. 3): Somatic ER tubule disruption causes enlarged mature lysosomes with reduced motility.

a-f Parameters indicated in each graph were quantified from DIV6 neurons transfected at DIV4 with LAMP1-GFP and a fill, together with control pSuper plasmid (n=14) or pSuper plasmids containing shRNAs targeting RTN4 plus DP1(n=14) (**a**), or together with SBP-RTN4 in absence (n=19) or presence of Strep-KIFC1 (n=17) or KIF5A-Strep (n=18) (**b-f**).

g-h Representative still images of the soma of DIV7 neurons transfected at DIV3 with a control pSuper plasmid (left panels) or pSuper plasmids containing shRNAs targeting RTN4 plus DP1 (right panels) together with LAMP1-GFP and a fill, and labelled live for active cathepsin-D and B (magenta) with SirLyso (**g**) and MagicRed (**h**), respectively. LAMP1 in green. Intensity profile line of a region of the soma for control and RTN4 plus DP1 knockdown neurons, on the right of each merged image.

i Representative still images and magnified region from soma of DIV7 neurons transfected with CD63-GFP (green) and pSuper plasmids containing shRNAs targeting RTN4 plus DP1 (top) or transfected with LAMP1-GFP (green) and RTN4-SBP in the presence of KIF5A-Strep (bottom), and labelled with SirLyso (magenta) prior to imaging.

j Quantification of normalized SirLyso signal intensity from somas of DIV6 neurons transfected with LAMP1-GFP and mCherry fill together with a control pSuper plasmid (n=21) or pSuper plasmids containing shRNAs targeting RTN4 plus DP1 (n=22).

k Quantification of protein levels of several lysosomal markers including LAMP1, LAMP2, Cathepsin B and D in neurons transfected with a control pSuper plasmid or pSuper plasmids containing shRNAs targeting RTNs or DP1. Dot plots show the average TMT ratios of pSuper versus untransfected neurons and RTNs shRNAs (left plot, blue color scale) or DP1 shRNA (right plot, red color scale) versus pSuper transfected neurons. The size and color of each dot reflects the average TMT ratio as indicated in the legend.

l Representative image and correlative light electron microscopy (CLEM) of lysosomes from somas of DIV8 neurons transfected with LAMP1-GFP (green) plus control pSuper plasmid and labeled with SirLyso (magenta) prior imaging. Several lysosomes were selected (shown with white arrowheads) for 3D-EM analyses. Reconstructed FIB. SEM slice of ROI in same (XY) orientation as FM, marked with yellow, blue and red lines that correspond to the orthogonal images shown on

right panel. XZ plane images corresponding to the yellow line, blue line and red line, indicating position of lysosomes showed in (**l**) and (**m**). Data are representative of three experiments. Boxplot shows 25/75- percentiles; the median; whiskers represent min to max values and individual datapoints each represent a neuron, in (**a**), (**b**), (**c**), (**d**), (**e**), (**f**) and (**j**) ns-not significant, *p<0.05, ***p<0.001 and comparing conditions to control (two-sided unpaired t test) in (A; p=0,0146), (Kruskal-Wallis test followed by Dunn's multiple comparison test) in (**b**;) and (two-sided Mann-Whitney test was performed) in (**j**). Scale bars represent 5 µm in (**g**), (**h**), (**i**) and (**l**, left top panel), 1 µm (**i**, enlarged images from orange boxes), (**l**, left bottom and right panels). Source data and exact p values are provided as a Source Data file.

Supplementary Fig. 4

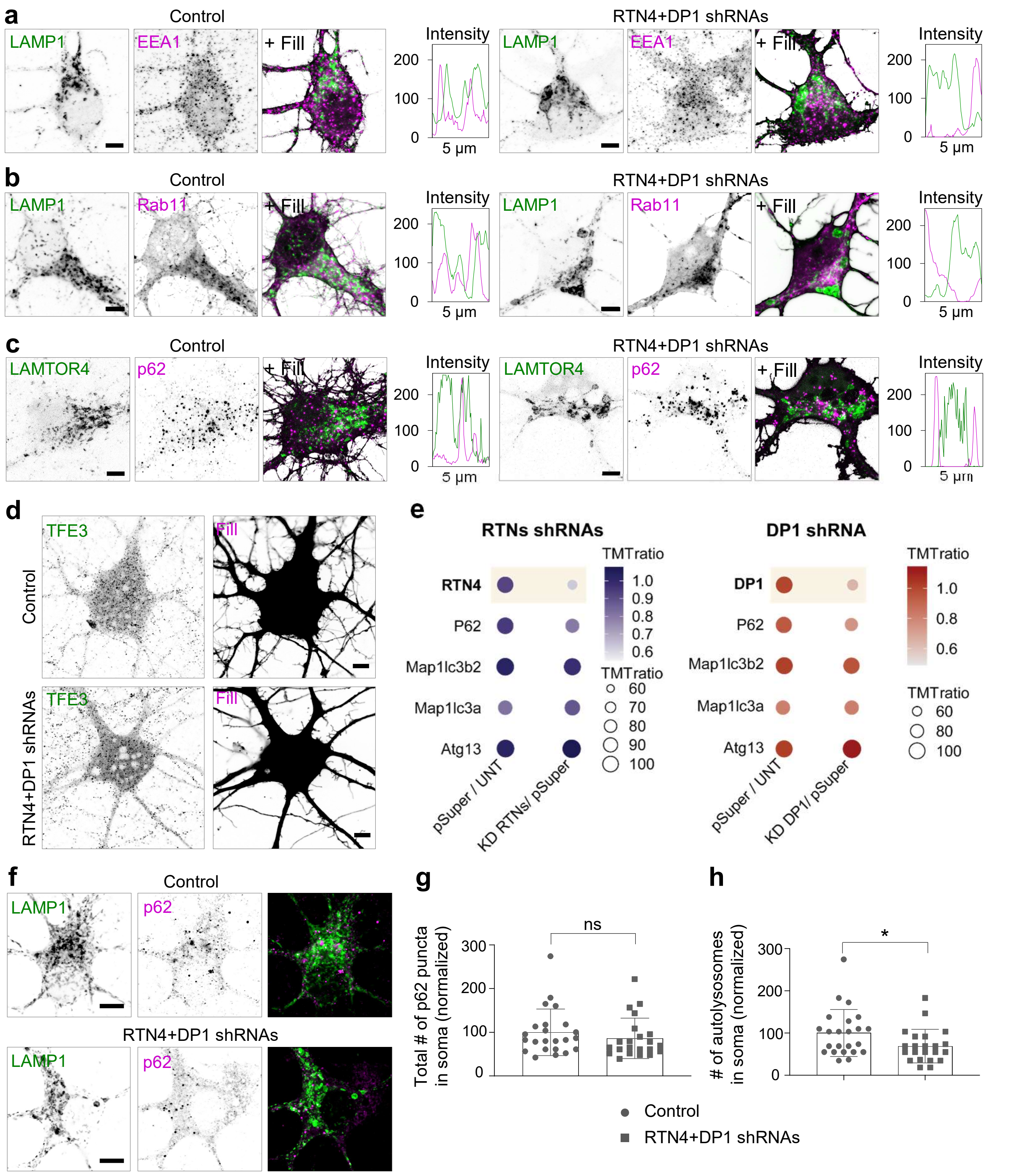

Supplementary Fig. S4 (related to Fig. 4): Endo-lysosomal markers are not particularly enriched in enlarged mature lysosomes, and mTORC1 signaling substrate-TFE3 and autophagy are not affected after ER tubule disruption.

a-c Representative images of lysosomes distributed in the somas of DIV7 neurons transfected at DIV3 with fill and a control pSuper plasmid (left panels) or pSuper plasmids containing shRNAs targeting RTN4 plus DP1 (right panels) together with LAMP1-GFP (green) or LAMP1- RFP (green) plus GFP-Rab11 (magenta) (**b**). Neurons were stained for EEA1 (early endosome marker, magenta) (**a**), or LAMTOR4 (lysosome marker, green) and p62 (autophagosome marker, magenta) (**c**). Intensity profile lines for different markers on the right of each merged image. Scale bars represent 5 μm in (**a**), (**b**) and (**c**). Data are representative of four experiments.

d Representative images of the soma from DIV8 neurons transfected with a fill and a control pSuper plasmid or pSuper plasmids containing shRNAs targeting RTN4 plus DP1 and labeled with an antibody against TFE3. From 141 and 124 neurons analyzed in each condition, none of them showed nuclear TFE3 signal. Data are representative of three experiments

e Quantification of protein levels of several autophagy-related proteins, including p62, LC3, Atg13, in neurons transfected with a control pSuper plasmid or pSuper plasmids containing shRNAs targeting RTNs or DP1. Dot plots show the average TMT ratios of pSuper versus untransfected neurons and RTNs shRNAs (left plot, blue color scale) or DP1 shRNA (right plot, red color scale) versus pSuper transfected neurons. The size and color of each dot reflects the average TMT ratio as indicated in the legend.

f Representative images of the soma from DIV8 neurons transfected with LAMP1-GFP and a control pSuper plasmid or pSuper plasmids containing shRNAs targeting RTN4 plus DP1 and labeled with an antibody against p62.

g-h Quantification of the total number of p62 positive vesicles (normalized) in somas (**g**) and quantification of the number of autolysosomes (normalized) in somas (**h**) from neurons indicated in (**f**; n=23 and n=22; respectively). Data are presented as mean values +/- SD and individual datapoints each represent a neuron, in (**g**) and (**h**); ns-not significant, $*p<0.05$ and comparing conditions to control (two-sided Mann-Whitney test was performed) in (**g**) and (**h**). Source data and exact p values are provided as a Source Data file.

Scale bars represent 5 μm in (**a**), (**b**), (**c**), (**d**) and (**f**).

Supplementary Fig. 5

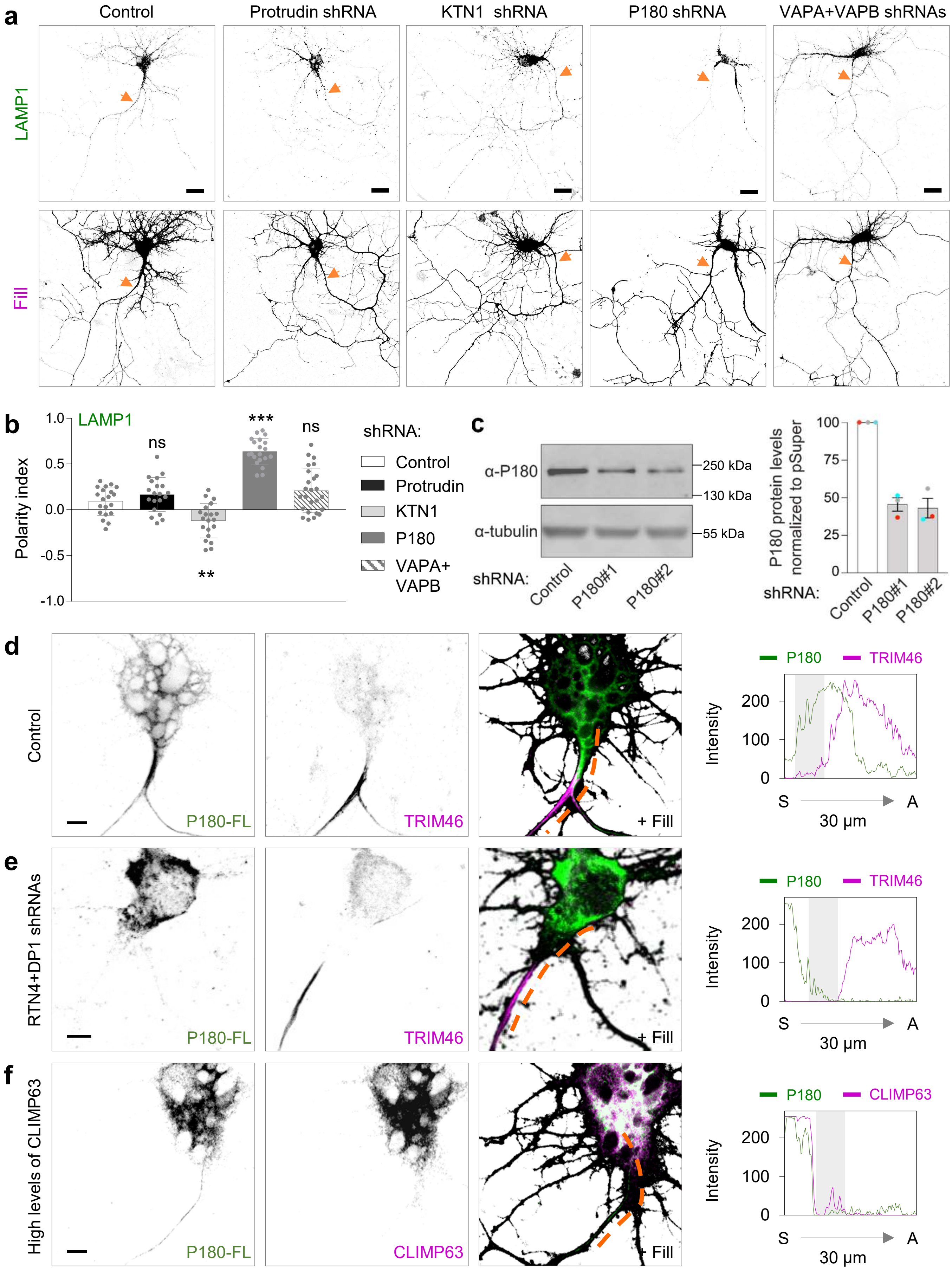

Supplementary Fig. 5 (related to Fig. 6): Lysosome distribution is only affected under the knockdown of P180, which is excluded from the pre-axonal region under knockdown of RTN4 plus DP1.

a-b Representative images of lysosome distribution in DIV7 neurons transfected with fill, LAMP1-GFP and pSuper plasmid or pSuper plasmids containing shRNA sequences targeting three kinesin-1-binding ER-proteins (protrudin, KTN1 and P180) as well as the tethering proteins VAPA and VAPB. Quantification of LAMP1 polarity indices in (**b**) (n=20, n=20, n=20, n=20 and n=25 for conditions shown in **a**; respectively).

c Western blot analysis of whole cell lysates from rat INS-1 cells transfected with a control pSuper plasmid or pSuper plasmids containing two different rat shRNA sequences targeting P180 (#1 or #2) and blotted against P180 and α-tubulin. The graph shows individual datapoints of three independent experiments and depicts P180 protein levels normalized to the pSuper control condition.

d-f Representative images of P180 distribution in the soma and pre-axonal region of DIV7 neurons transfected with fill and P180-GFP (green in merges) together with pSuper plasmid (**d**) or pSuper plasmids containing shRNAs targeting RTN4 plus DP1 (**e**) or high expression of the cisternae-shaping protein CLIMP63-RFP (magenta) (**f**). Axon initial segment was labelled with an antibody against TRIM46 (magenta in merges) in (**d**) and (**e**). Intensity profile lines from the soma to the proximal axon, indicated with orange lines in merged images, are shown in the right panels. Data are representative of three experiments.

Scale bars in images represent 5 μm (**a**), (**d**), (**e**), (**f**) and orange arrows point to the proximal axon in (**a**). Data are presented as mean values +/- SD and individual datapoints each represent a neuron; ns-not significant, $^{**}p<0.01$ and $^{***}p<0.001$ comparing conditions to control (ordinary one-way ANOVA followed by a Dunnet's multiple comparison) in (**b**). Source data and exact p values are provided as a Source Data file.

Supplementary Fig. 6

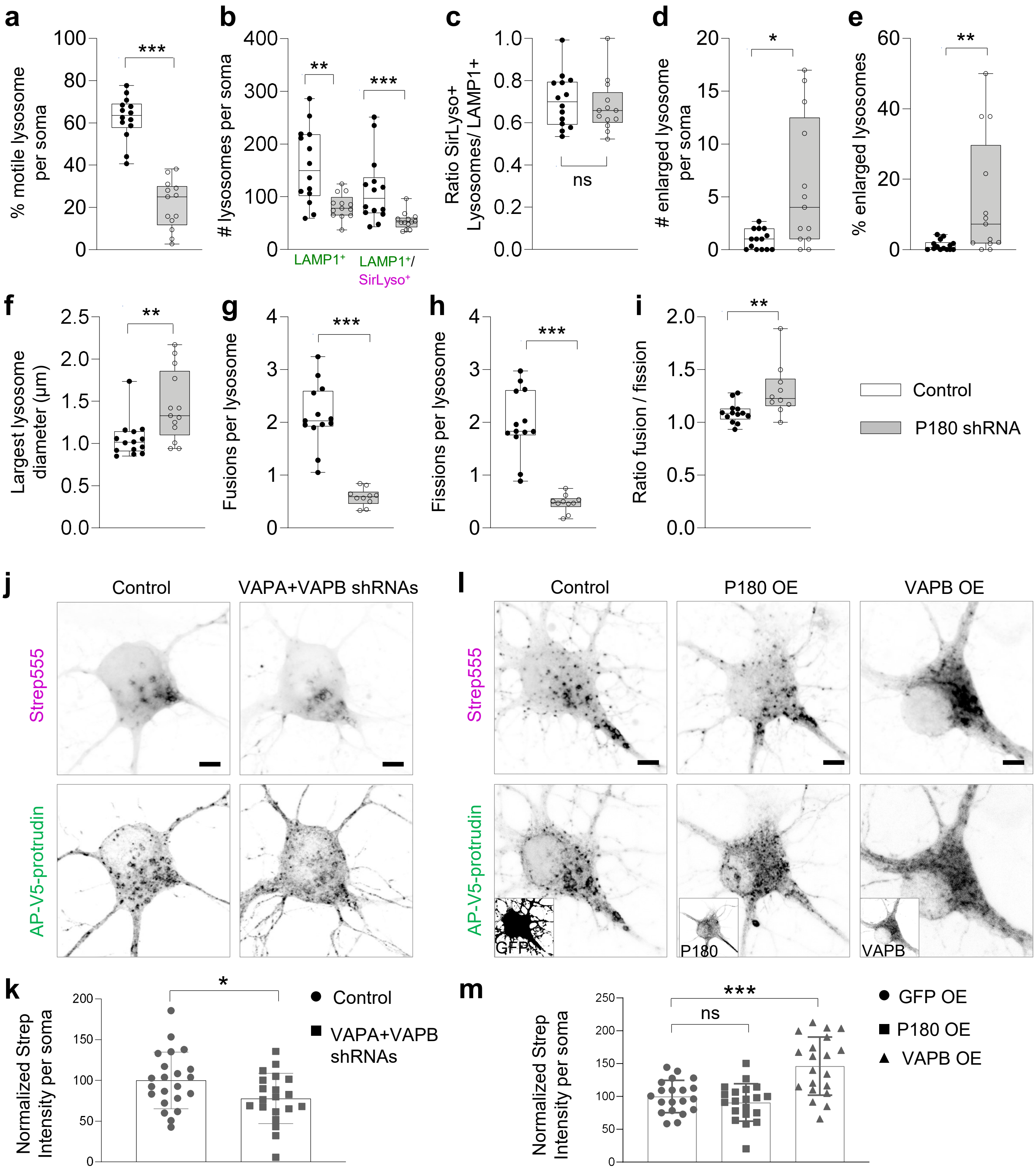

Supplementary Fig. 6 (related to Fig. 5 and Fig. 6): P180 is important for lysosome fission but not for ER-lysosome contacts.

a-i Parameters indicated in each graph were quantified from neurons transfected with a fill, LAMP1-GFP and a control pSuper plasmid (n=14 for **a-f**; n=13 for **g-i**) or pSuper plasmids containing shRNAs targeting P180 (n=13 for **a-f**; n=10 for **g-i**).

j Representative images of the soma from DIV8 neurons transfected at DIV4 with AP-V5-Protrudin and EX-HA-Rab7 together with a control pSuper plasmid or pSuper plasmids containing shRNA sequences targeting VAPA plus VAPB and labeled with an antibody against Alexa-555 conjugated Strep.

k Normalized average Strep intensity in the soma of neurons transfected as in (**j** n=20 and 22, respectively).

l Representative images of the soma from DIV7-8 neurons transfected at DIV3-4 with AP-V5-Protrudin and EX-HA-Rab7 together with GFP fill or P180-GFP or VAPB-GFP and labeled with an antibody against Alexa-555 conjugated Strep. OE stands for overexpression.

m Normalized average strep intensity in the soma of neurons transfected as in (**l**). n=20 neurons per condition.

Scale bars represent 5 μm in (**j**) and (**l**). Individual datapoints each represent a neuron in (**a**-**i**, **k** and **m**) Boxplot shows 25/75- percentiles, the median and whiskers represents min to max, individual datapoints each represent a neuron in (**a-i**). Data are presented as mean values +/- SD in (**k**) and (**m**); ns- not significant, *$p<0.05$ **$p<0.01$ and ***$p<0.001$ comparing conditions to control (two-sided Mann-Whitney test) in (**b-i**), (two-sided unpaired t test) in (**a** and **k**) and (one-way ANOVA test followed by a Sidak's multiple comparisons test) in (**m**). Source data and exact p values are provided as a Source Data file.

Supplementary Table: DNA primers list

Name	Sequence
HA-KIF5-Strep FW primer	5'ccggtgcccaccatgtacccatacgatgttcctgactatgcgggctatccctatgacgtcccgga ctatgcaggatcctatccatatgacgttccagattacgctggatccgt -3'
HA-KIF5-Strep Rev primer	5'gtacacggatccagcgtaatctggaacgtcatatggataggatcctgcatagtccgggacgtcat agggatagcccgcatagtcaggaacatcgtatgggtacatggtgggca-3'
mcherry-P180 FW primer	5'-agcgctaccggactcagatctcgagcaccatggatatttacgacactcaaaccttgg-3'
mcherry-P180 Rev primer	5'-caccatggtggcgaccggtggatccgggctaccgctgccgctacccacgctggtgccctcctt-3'
P180ΔKIF5-BD-GFP fragment 1 FW primer	5'-agcgctaccggactcagatctcgagcaccatggatatttacgacactcaaaccttg ggggttgtgg-3'
P180ΔKIF5-BD-GFP fragment 1 Rev primer	5'-ccgctgccgctacctgcggcgcccaccttggc-3'
P180ΔKIF5-BD-GFP fragment 2 FW primer	5'-gggcgccgcaggtagcggcagcggtagcgagcaggaccccgttcagctg-3'
P180ΔKIF5-BD-GFP fragment 2 Rev primer	5'caccatggtggcgaccggtggatccgggctaccgctgccgctacccacgctggtgccctcctt-3'
Split-AP-V5-C1 FW primer for AP	5'-cgtcagatccgctagcgctaccggtgccaccatggggaaatcatacccaacag-3'
Split-AP-V5-C1 Rev primer for AP	5'-tgccgctaccttccttctctccactcag-3'
Split-AP-V5-C1 FW primer for V5	5'-agagaaggaaggtagcggcagcggtagc-3'
Split-AP-V5-C1 Rev primer for V5	5'-aattcgaagcttgagctcgagatctgctaccgctgccgctaccggtgctgtccaggccc-3'
Split-AP-V5-protrudin FW primer	5'-agcggcagcggtagcagatctcgagccatgcagacatcagaacgtg-3'
Split-AP-V5-protrudin Rev primer	5'-gcggtaccgtcgactgcagaattctcacttgctcaaggtctg-3'
Split-EX-HA3x-C1 FW primer for EX	5'-cgtcagatccgctagcgctaccggtgaaccatggggctgctgc-3'
Split-EX-HA3x-C1 Rev primer for EX	5'-cgtatgggtagctaccgctgccgctaccggcgtcggcaaatcccag-3'
Split-EX-HA3x-C1 FW primer for HA	5'-tgccgacgccggtagcggcagcggtagctacccatacgatgttcctg-3'
Split-EX-HA3x-C1 Rev primer for HA	5'-aattcgaagcttgagctcgagatctagcgtaatctggaacgtc-3'
Split-EX-HA3x-Rab7 FW primer	5'-gttccagattacgctagatctcgagccggtagcggcagcggtagcatgacc tctaggaagaaagtg-3'
Split-EX-HA3x-Rab7 Rev primer for	5'-cgcggtaccgtcgactgcagaattctcagcaactgcagctttc-3'
Split-EX-HA3x-Rab7a-T22N FW primer	5'-ggagattctggagttggtaagaattcactcatgaaccagtatgtg-3'
Split-EX-HA3x-Rab7a-T22N Rev primer	5'-cacatactggttcatgagtgaattcttaccaactccagaatctcc-3'
GB-V5-C1 FW primer for GB	5'- tcagatccgctagcgctaccggtgccaccatggcctcgagagcctcc-3'

GB-V5-C1 Rev primer for GB	5’- gccgctacccttgtaccgctcgtccatg-3’
GB-V5-C1 FW primer for V5	5’-cggtacaagggtagcggcagcggtagc-3’
GB-V5-C1 Rev primer for V5	5’- agatctgagtccggacttgtacacgtagaatcgagaccgaggagag-3’
GB-V5-Rab7 FW primer	5’- ttctacgtgtacaagtccggactcagatctcggtagcggcagcggtag-3’
GB-V5-Rab7 Rev primer	5’- ggtggatcccgggcccgcggtaccgtcgactcagcttcttctagattttgaatgctgaataagc-3’
RA-HA-C1 FW primer for RA	5’- tcagatccgctagcgctaccggtgccaccatggtgagcaagagcgag-3’
RE-HA-C1 Rev primer for RA	5’- gccgctacccttgtacagctcgtccatg-3’
RA-HA-C1 FW primer for HA	5’- ctgtacaagggtagcggcagcggtagc-3’
RA-HA-C1 Rev primer for HA	5’- agatctgagtccggacttgtacaagcgtaatctggaacgtcatatggatag-3’
RA-HA-protrudin FW primer	5’- tacaagtccggactcagatctcgagatgcagacatcagaacgtg-3’
RA-HA-protrudin Rev primer	5’- gcggtaccgtcgactgcagaattctcacttgctcaaggtctg-3’
