# Supplementary material for: ER – lysosome contacts at a pre-axonal region regulate axonal lysosome availability
Source: Nat Commun. 2021 Jul 23;12:4493. doi: 10.1038/s41467-021-24713-5 (PMC8302662; doi:10.1038/s41467-021-24713-5)
Supplement: Supplementary file 3 — Description of Additional Supplementary Files [file 41467_2021_24713_MOESM3_ESM.pdf]

Description of Additional Supplementary Files

File Name: Supplementary Movie 1
Description: **(related to Fig. 1 and Supplementary Fig. 1): ER nanoscale structure is altered by knockdown of RTN4 and DP1.** Volumetric rendering of nanoscale ER structure after Ten-fold Robust Expansion microscopy (TREx) (Damstra et al., 2021; BioRxiv) of the soma from neurons transfected with Sec61β-GFP, together with control pSuper (left panel) or shRNAs targeting RTN4 plus DP1 (right panel).

File Name: Supplementary Movie 2
Description: **(related to Fig. 1): ER tubule disruption under RTN4 plus DP1 knockdown.** Dynamics of ER at the pre-axonal region of neurons transfected with Sec61-GFP (green), a fill and control pSuper (top) or shRNAs targeting RTN4 plus DP1 (bottom) and labeled with NF (magenta). Right panels show higher magnification of the pre-axonal region. Notice dynamic ER tubules at the pre-axonal region and along the axon in control cells, and impaired ER tubule formation and entrance into the pre-axonal region under knockdown of RTN4 plus DP1. Neurons were recorded every 1 sec for 300 sec.

File Name: Supplementary Movie 3
Description: **(related to Fig. 1): ER tubule disruption impairs lysosome translocation into the axon.**
Transport of LAMP1-positive lysosomes in proximal axons of DIV7 neurons co-transfected with LAMP1-GFP, fill and control pSuper (top) or shRNAs targeting RTN4 plus DP1 (bottom). Endogenous NF marks the axon initial segment. Neurons were recorded every 1 sec for 300 sec. Orange dashed lines in left panels indicate region of the straightened proximal axon showed in right panels.

File Name: Supplementary Movie 4
Description: **(related to Fig. 2): Somatic ER tubule repositioning into the axon impairs axonal lysosome translocation.** Transport of LAMP1-positive lysosomes in proximal axons of DIV7 neurons transfected with only LAMP1-RFP (magenta) and GFP-SBP-RTN4 (green) plus fill as a control (left panel), or together with Strep-KIFC1 (middle panel) to pull ER tubules from axon to soma, or KIF5A-Strep (right panel) to pull ER tubules from soma to axon. Neurons were imaged every 1 sec for 300 sec.

File Name: Supplementary Movie 5
Description: **(related to Fig. 3): Enlarged and less motile lysosomes in the soma of neurons after ER tubule disruption.** Lysosome motility in the soma of DIV7 neurons co-transfected with LAMP1-GFP, fill and control pSuper (top) or shRNAs targeting RTN4 plus DP1 (bottom). Neurons were recorded every 1 sec for 300 sec.

File Name: Supplementary Movie 6
Description: **(related to Fig. 3): Somatic ER tubule repositioning into the axon impairs lysosome size, motility and translocation into the axon.** Part I: Dynamics of LAMP1-positive lysosomes in the soma of DIV6 neurons transfected on DIV4 with only LAMP1 (green) and SBP-RTN4A plus fill (blue) as a control (left) or together with Strep-KIFC1 (middle) or KIF5A-Strep (right). Part II: Somatic ER tubule disruption causes reduced motility of enlarged lysosomes in a pre-axonal region, thereby impairing axonal translocation (NF in magenta). Neurons were recorded every 1 sec for 300 sec.

File Name: Supplementary Movie 7
Description: **(related to Figs. 3 and 4): Motile mature and immature lysosomes in the soma of control neurons and enlarged and less motile mature lysosomes in the soma after ER-tubule disruption.** Neurons were imaged every 1 sec for 300 sec.

File Name: Supplementary Movie 8
Description: **(related to Fig. 4): Lysosome fusion and fission events and impaired fission after ER tubule disruption.** Movies of fusion and fission events from still images shown in Figure 4.

File Name: Supplementary Movie 9
Description: **(related to Fig. 6): Knockdown of P180 leads to the accumulation of enlarged lysosomes at a pre-axonal region, thereby impairing axonal lysosome translocation.** Lysosome motility from still image shown in Figure 6C. Neuron was recorded every 1 sec for 300 sec. AIS labelled with NF (green) shown in green dashed box. Enlarged and less motile lysosomes (magenta) in a pre-axonal region is shown in orange dashed box.

File Name: Supplementary Movie 10
Description: **(related to Fig. 6): Enlarged mature lysosomes show reduced motility and accumulate in a pre-axonal region after P180 knockdown.** Motility of lysosomes in the soma and pre-axonal region of neurons after P180 knockdown. Neurons were recorded every 1 sec for 300 sec. Compare with control neurons shown in Supplementary Movie 6.

File Name: Supplementary Movie 11
Description: **(related to Fig. 7): Lysosome dynamics at the site of ER tubule – lysosome contact in the pre-axonal region.** Part I: Lysosome dynamics associated to ER tubules at the pre-axonal region from stills shown in Figure 7A. Neurons were recorded every 1.5 sec for 120 sec. Part II: Dynamics of lysosome – ER contacts and associated lysosome fissions at the pre-axonal region using GB-RA contact assay, from still images shown in Figures 7C and 7D. Neurons were recorded every 1.5 sec for 120 sec (left top and left middle panels) and every 1 sec for 120 sec (left bottom and right panels).
