# Supplementary material for: ER – lysosome contacts at a pre-axonal region regulate axonal lysosome availability
Source: Nat Commun. 2021 Jul 23;12:4493. doi: 10.1038/s41467-021-24713-5 (PMC8302662; doi:10.1038/s41467-021-24713-5)
Supplement: Supplementary file 15 — Reporting Summary [file 41467_2021_24713_MOESM15_ESM.pdf]

nature research

Corresponding author(s): Ginny G. Farias

Last updated by author(s): Jun 14, 2021

# Reporting Summary

Nature Research wishes to improve the reproducibility of the work that we publish. This form provides structure for consistency and transparency in reporting. For further information on Nature Research policies, see our Editorial Policies and the Editorial Policy Checklist.

## Statistics

For all statistical analyses, confirm that the following items are present in the figure legend, table legend, main text, or Methods section.

| n/a | Confirmed | |
|---|---|---|
| ☐ | ☒ | The exact sample size ($n$) for each experimental group/condition, given as a discrete number and unit of measurement |
| ☐ | ☒ | A statement on whether measurements were taken from distinct samples or whether the same sample was measured repeatedly |
| ☐ | ☒ | The statistical test(s) used AND whether they are one- or two-sided<br>*Only common tests should be described solely by name; describe more complex techniques in the Methods section.* |
| ☐ | ☒ | A description of all covariates tested |
| ☐ | ☒ | A description of any assumptions or corrections, such as tests of normality and adjustment for multiple comparisons |
| ☐ | ☒ | A full description of the statistical parameters including central tendency (e.g. means) or other basic estimates (e.g. regression coefficient) AND variation (e.g. standard deviation) or associated estimates of uncertainty (e.g. confidence intervals) |
| ☐ | ☒ | For null hypothesis testing, the test statistic (e.g. $F$, $t$, $r$) with confidence intervals, effect sizes, degrees of freedom and $P$ value noted<br>*Give P values as exact values whenever suitable.* |
| ☒ | ☐ | For Bayesian analysis, information on the choice of priors and Markov chain Monte Carlo settings |
| ☒ | ☐ | For hierarchical and complex designs, identification of the appropriate level for tests and full reporting of outcomes |
| ☒ | ☐ | Estimates of effect sizes (e.g. Cohen's $d$, Pearson's $r$), indicating how they were calculated |

*Our web collection on statistics for biologists contains articles on many of the points above.*

## Software and code

Policy information about availability of computer code

| | |
|---|---|
| Data collection | All fixed cell imaging was performed using a Zeiss LSM 700 confocal microscope with Zen imaging software (Zeiss) version 8.1.7.484. For CLEM imaging, samples were imaged using a Scios Dualbeam FIB-SEM under high vacuum conditions and slice&view v3 software was used for automated serial imaging (Thermo Fisher Scientific; v3). All live imaging experiments were performed using a i Nikon Eclipse Ti-E inverted microscope with MetaMorph (Molecular Devices) version 7.10.2.240 software for controlling all devices. For expansion microscopy, images were acquired using a Leica TCS SP8 STED 3X microscope equipped with a HC PL APO 86x/1.20W motCorr STED (Leica 15506333) water objective and images were deconvolved with Huygens Professional (SVI) version 20.04. Western blots were scanned on Odyssey Clx imaging system (LICOR) with Image Studio version 5.2 software. |
| Data analysis | Image processing and analysis was performed using ImageJ 1x/FIJI software. For expansion microscopy, images were processed using ImageJ 1.53c and Arivis 3.4. Microsoft Excel was used for data collection and Graphpad Prism 8 software was used to perform statistical analysis and to display data. Dot blots were generated in R software. No custom codes were used in this study. |

For manuscripts utilizing custom algorithms or software that are central to the research but not yet described in published literature, software must be made available to editors and reviewers. We strongly encourage code deposition in a community repository (e.g. GitHub). See the Nature Research guidelines for submitting code & software for further information.

## Data

Policy information about availability of data

All manuscripts must include a data availability statement. This statement should provide the following information, where applicable:

- Accession codes, unique identifiers, or web links for publicly available datasets
- A list of figures that have associated raw data
- A description of any restrictions on data availability

The datasets generated during and/or analysed during the current study are available in the data availability section in the manuscript.
Quantitative proteomics dataset from Farías et al., 2019 (ProteomeXchange dataset PXD012264):
http://proteomecentral.proteomexchange.org/cgi/GetDataset?ID=PXD012264-2&test=no

# Field-specific reporting

Please select the one below that is the best fit for your research. If you are not sure, read the appropriate sections before making your selection.

☒ Life sciences ☐ Behavioural & social sciences ☐ Ecological, evolutionary & environmental sciences

For a reference copy of the document with all sections, see nature.com/documents/nr-reporting-summary-flat.pdf

# Life sciences study design

All studies must disclose on these points even when the disclosure is negative.

| | |
|---|---|
| Sample size | Sample sizes were not pre-determined. We chose samples sizes based on previously observed variation in imaging of our primary cultures of neurons. Because of the nature of primary neuron cultures, neurons might have variation in their growth rate, morphology, or expression levels. To eliminate the effects from these variations, we used at least 20 neurons per quantifications from fixed cells' imaging and at least 10 neurons for quantifications from live cell imaging. Similar sample sizes have been reported in other publications (Farias et al., 2015; Tortosa et al., 2017; Farias et al., 2017; Farias et al 2019; Pan et al., 2019; De Pace et al., 2020) |
| Data exclusions | No data were excluded from analyses. |
| Replication | All experiments were repeated at least two-three times from independent primary cultures, except for correlative EM (CLEM) and expansion microscopy. For CLEM at least 6 images were obtained for light microscopy and at least three of these images were processes for correlation between light and EM microscopy from the same neuron culture but for different neurons. Representative image are shown, and there is no quantification associated to CLEM. All biological and technical replications were successful and gave similar results. For expansion microscopy, 4 images with the same quality and same results were obtained from the same neuron culture but from different neurons. Representative images are shown, and there is no quantification associated with expansion microscopy |
| Randomization | Randomization is not relevant for our experiments as samples were not allocated into groups. |
| Blinding | Investigators were not blinded for data collection and data analysis. Since most of the experimental procedures and data analyses were performed by more than one researcher obtaining similar results and all the data analysis were performed by using softwares, blinding was not relevant for this study. |

# Reporting for specific materials, systems and methods

We require information from authors about some types of materials, experimental systems and methods used in many studies. Here, indicate whether each material, system or method listed is relevant to your study. If you are not sure if a list item applies to your research, read the appropriate section before selecting a response.

## Materials & experimental systems

| n/a | Involved in the study | |
|---|---|---|
| ☐ | ☒ | Antibodies |
| ☐ | ☒ | Eukaryotic cell lines |
| ☒ | ☐ | Palaeontology and archaeology |
| ☐ | ☒ | Animals and other organisms |
| ☒ | ☐ | Human research participants |
| ☒ | ☐ | Clinical data |
| ☒ | ☐ | Dual use research of concern |

## Methods

| n/a | Involved in the study | |
|---|---|---|
| ☒ | ☐ | ChIP-seq |
| ☒ | ☐ | Flow cytometry |
| ☒ | ☐ | MRI-based neuroimaging |

## Antibodies

Antibodies used

rabbit anti-LAMTOR4 (Cell Signaling, clone D6A4V, Cat# 12284S, RRID: AB_2797870),
mouse anti-EEA1 (BD Biosciences, Cat# 610456, RRID: AB_397829),
mouse anti-P62 (Abcam, Cat# 56416, RRID:AB_945626),
mouse anti-V5 (Thermo Fisher Scientific Cat# R960-25, RRID:AB_2556564),
rat anti-HA (Roche Cat# 11867423001, RRID:AB_390918),
mouse anti-Pan-Neurofascin external (clone A12/18; UC Davis/NIH NeuroMab, Cat# 75-172, RRID: AB_2282826),
rabbit anti TFE-3 (Cell Signaling, Cat#14779, RRID:AB_2687582),
mouse anti p62/ SQSTM1 (Abnova, Cat# H00008878-M01, RRID:AB_437085),
rabbit anti-TRIM46; homemade (van Beuningen et al., 2015),
rabbit anti RRBP1(P180)(Abcam; Cat#ab95983, RRID:AB_10678752),
mouse-anti-GFP (Thermo Fisher Scientific Cat# A-11120; RRID:AB_221568),
mouse anti alpha tubulin (Sigma, clone B-5-1-2,Cat#T5168, RRID:AB_477579)
Streptavidin, Alexa Fluor 555 conjugate (Thermo Fisher Scientific Cat# s21381, RRID: AB_2307336),
Streptavidin, Alexa Fluor 568 conjugate (Thermo Fisher Scientific Cat# S-11226, RRID:AB_2315774),
donkey anti-mouse Alexa488 (Molecular Probes, Cat# A21202, RRID: AB_141607),
donkey anti-mouse Alexa555(Molecular Probes, Cat# A31570, RRID: AB_2536180),
donkey anti-mouse Alexa647 (Molecular Probes, Cat#A31571, RRID: AB_162542),
donkey anti-rabbit Alexa488 (Molecular Probes, Cat# A21206, RRID: AB_141708),
donkey anti-rabbit Alexa555 (Molecular Probes, Cat# A31572, RRID: AB_162543),
donkey anti-rabbit Alexa647 (Molecular Probes, Cat# A31573, RRID: AB_2536183),
goat anti-mouse Alexa405 (Molecular Probes, Cat# A31553, RRID: AB_221604),
goat anti-Mouse IgG (H+L) Highly Cross-Adsorbed Alexa Fluor 488 (Thermo Fisher Scientific Cat# A-11029, RRID:AB_2534088),
goat anti-rabbit Alexa405 (Molecular Probes, Cat# A31556; RRID: AB_221605),
goat anti-rat Alexa 488 (Thermo Fisher Scientific Cat# A-11006, RRID:AB_2534074),
goat anti-rat Alexa 568 (Thermo Fisher Scientific Cat# A-11077, RRID:AB_2534121),
IRDye 680RD goat anti mouse IgG (LI-COR Biosciences, Cat#926-68070, RRID:AB_10956588),
IRDye 800CW goat anti rabbit IgG ( LI-COR Biosciences,Cat#926-32211, RRID:AB_62843).

Validation

Rabbit anti-LAMTOR4 (Cell Signaling, clone D6A4V, Cat# 12284S, RRID: AB_2797870): manufacturer's website noted that LAMTOR4/C7orf59 (D6A4V) Rabbit mAb recognizes endogenous levels of total LAMTOR4/C7orf59 protein and has reactivity for human, mouse, rat and monkey. Some of the relevant citations are Jia R et al., 2019; Janssen AF et al., 2018; Sun J et al., 2018. Data provided in manuscript in Figure 1c, 3b, 5b, 5f, 5h, S4c.
mouse anti-EEA1 (BD Biosciences, Cat# 610456, RRID: AB_397829): manufacturer's website noted that antibody was routinely tested for western blot, tested during development for immunofluorescence, and not recommended for immunohistochemistry and immunoprecipitation and has reactivity for rat (QC testing), human, dog, chicken (tested in development). Data provided in the manuscript in supplementary Figure S4a.
mouse anti-P62 (Abcam, Cat# 56416, RRID:AB_945626) : manufacturer's website noted that antibody was suitable for IHC-P, WB, ICC/IF, Flow Cyt, IHC-Fr and has reactivity for mouse, rat, human, rhesus monkey. Some of the relevant citations are Zhang J et al., 2020, Zhu N et al., 2020, Chen X et al, 2020. Data provided in the manuscript in supplementary Figure 4f and S4c.
mouse anti-V5 (Thermo Fisher Scientific Cat# R960-25, RRID:AB_2556564): manufacturer's website noted that antibody was tested for Elisa, ICC, IF, IP and WB and interact with bacteria, bovine, C. elegans, fruit fly, hamster, human, insect, marsupial, mouse, non-human primate, pig, protozoa, pat, tag, virus, yeast. This antibody was cited in 874 publication and some of the relevant citations are Sales EC et al., 2019, Hickey SL et al., 2019, Ashley J et al., 2019. Data provided in Figure 5f and 5h.
rat anti-HA (Roche Cat# 11867423001, RRID:AB_390918),: manufacturer's website noted that antibody was suitable for dot blots, ELISA, immunocytochemistry, immunoprecipitation, western blots and cited in Elisabeth Stes et. al, 2014, Kroening et al, 2009, and Jung et al, 2015. Data provided in manuscript in Figure 5.
mouse anti-Pan-Neurofascin external (clone A12/18; UC Davis/NIH NeuroMab, Cat# 75-172, RRID: AB_2282826): manufacturer's website noted that antibody was tested for immunoblotting, immunohistochemistry and has reactivity for human and rat. Data provided in manuscript in Figure 6c, 7c and Videos S2, S3 and S9.
rabbit anti-TRIM46 (Homemade; validated in van Beuningen et al., 2015). Antibody has been used in several publications, including Tortosa et al., 2017; Farias et al., 2019; Pan et al., 2019; Harterink et al., 2019; Freal et al., 2019; Lindhout et al., 2021. Data provided in Figure 5i, S5d, S5e, S5f.
rabbit anti TFE-3 (Cell Signaling, Cat#14779, RRID:AB_2687582): manufacturer's website noted that antibody was tested for WB and interact with human, mouse and rat. Bordi et al, Autophagy, 2016 showed ICC localization of TFE3 in the hippocampal CA1 region and the nucleus shuttling of TFE3. Data provided in Figure S4d.
mouse anti p62/ SQSTM1 (Abnova, Cat# H00008878-M01, RRID:AB_437085): manufacturer's website noted that antibody was suitable for WB, IF, Sandwich ELISA, ELISA and has reactivity for human and mouse. Some of the relevant citations are Mu et al., 2021; He et al., 2021 and Nomura et al., 2021. Data is provided in Figure S4f.
rabbit anti RRBP1(P180)(Abcam; Cat#ab95983, RRID:AB_10678752): Tested in this study by WB. Manufacturer's website noted that antibody is suitable for WB, IHC-P and ICC/IF and has reactivity for mouse and human. Some of the relevant citations are Wang M et al., 2020; and Hung V et al., 2019. Data is provided in Figure S5c.
mouse anti alpha tubulin (Sigma, clone B-5-1-2,Cat#T5168, RRID:AB_477579):manufacturer's website noted that antibody was suitable for WB and has reactivity for bovine, rat, yeast, human, mouse, chicken, fungi, amphibian. This antibody was cited in 3124 publication. Data provided in Figure S5c
mouse-anti-GFP (Thermo Fisher Scientific Cat# A-11120; RRID:AB_221568:manufacturer's website noted that antibody was tested for WB, ELISA, immunoprecipitation (IP) and immunocytochemistry and has reactivity for C. elegans, Fruit fly, Human, Insect, Mouse, Rat, Tag, Zebrafish. Some of the relevant citations are Nyugen PM. et al, 2019; Jiang N et. al., 2019 and Yu B. et al., 2018. Data provided in FigureS1 and VideoS1.

## Eukaryotic cell lines

Policy information about cell lines

| | |
|---|---|
| Cell line source(s) | INS-1 rat insulinoma cell line, Clone 832/3 (SCC208), Merck. INS-1 832/3 is a derivative of INS-1 cells originally established from an x-ray induced insulinoma in rat1. The INS-1 832/3 cell line is a subclone of INS-1 that was stably transfected with a CMV promoter-human insulin expression plasmid carrying a geneticin (G418)-resistance marker for selection. |
| Authentication | Cells were verified by Merck to be of rat origin and negative for inter-species contamination from mouse, chinese hamster, Golden Syrian hamster, human and non-human primate (NHP) as assessed by a Cell Line Examination and Report (CLEAR) PCR Panel performed by Charles River Animal Diagnostic Services. |
| Mycoplasma contamination | INS-1 cell line tested negative for mycoplasma |
| Commonly misidentified lines (See ICLAC register) | Misidentified lines were not used in this study |

## Animals and other organisms

Policy information about studies involving animals; ARRIVE guidelines recommended for reporting animal research

| | |
|---|---|
| Laboratory animals | 2.5 months old female pregnant Wistar rats were obtained from Janvier, and embryos (both genders) at E18 stage of development were used for primary cultures of hippocampal neurons. The animals, pregnant females and embryos have not been involved in previous procedures. |
| Wild animals | This study did not involve wild animals |
| Field-collected samples | This study did not involve sample collected from the field |
| Ethics oversight | All experiments were approved by the DEC Dutch Animal Experiments Committee (Dier Experimenten Commissie), performed in line with institutional guidelines of University Utrecht, and conducted in agreement with Dutch law (Wet op de Dierproeven, 1996) and European regulations (Directive 2010/63/EU). The animal protocol has been evaluated and approved by the national CCD authority (license AVD1080020173404). This Information is provided in the manuscript. |

Note that full information on the approval of the study protocol must also be provided in the manuscript.
